# P-1412. Incidence of Mycobacterium tuberculosis Screening and Detection in People With Human Immunodeficiency Virus in Custody Within the Illinois Department of Corrections

**DOI:** 10.1093/ofid/ofaf695.1599

**Published:** 2026-01-11

**Authors:** Luke E Stickler, Emily N Drwiega, Daniel McKelvey, Tommy Windt, Scott Borgetti, Mahesh C Patel, Melissa E Badowski

**Affiliations:** University of Illinois Chicago, Retzky College of Pharmacy, Chicago, IL; University of Illinois Chicago, Chicago, Illinois; University of Illinois Retzky College of Pharmacy, Chicago, Illinois; University of Illinois Retzky College of Pharmacy, Chicago, Illinois; University of Illinois at Chicago, Chicago, Illinois; University of Illinois Chicago, Chicago, Illinois; University of Illinois Chicago, Chicago, Illinois

## Abstract

**Background:**

Tuberculosis (TB) screening is crucial for people in custody, given the close-quarters living. People with human immunodeficiency virus (PWH) in custody face a compounded risk, due to a decreased ability to defend against TB. This study investigated TB screening incidence, detection, & treatment (tx) practices of PWH in custody within the Illinois Department of Corrections (IDOC).

**Table 1:**
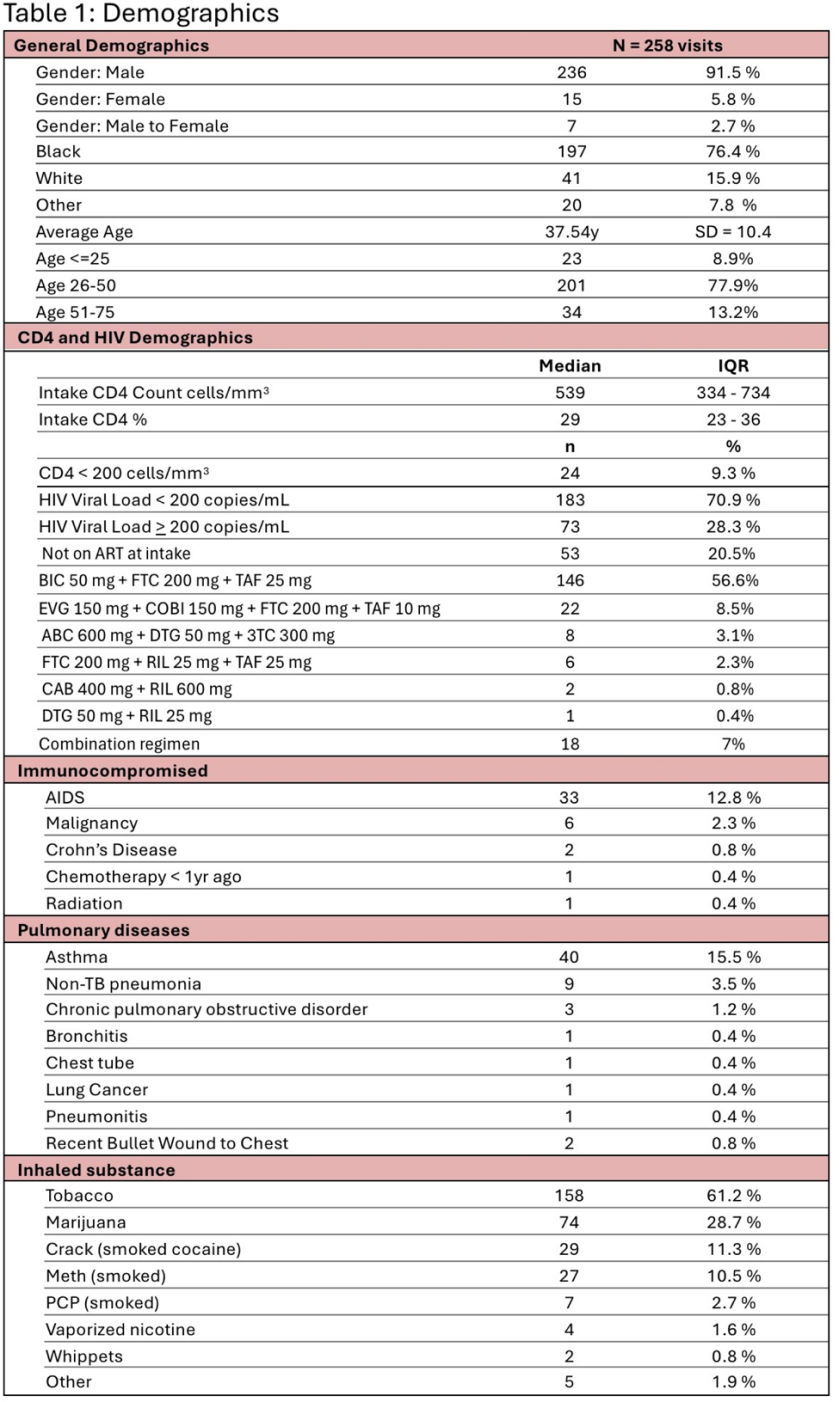
Demographics Table 1 depicts subject demographics such as gender, age, HIV RNA and CD4 information, and risk factors to having worse outcomes should patients acquire tuberculosis.

**Table 2:**
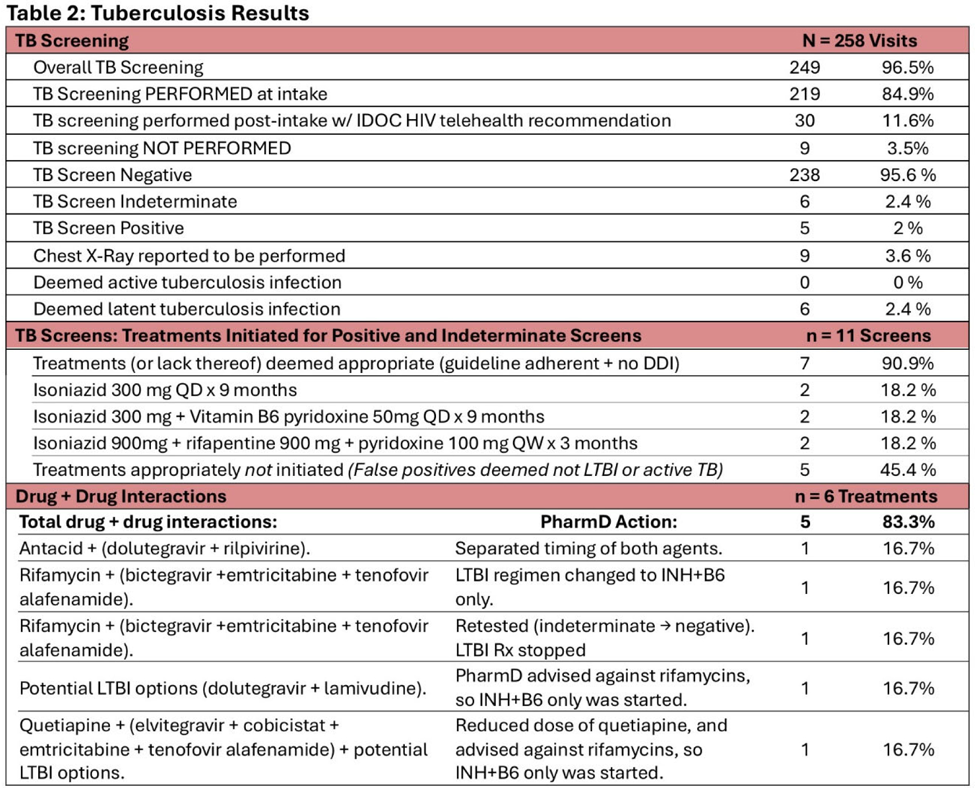
Tuberculosos Results Table 2 depicts how many subjects were screened for tuberculosis (TB), late TB screens, TB screen results, treatments, guideline-appropriateness of treatment, incidence of drug+drug interactions (DDI), and PharmD interventions to DDI.

**Methods:**

This was an IRB-approved, retrospective chart review of PWH serviced by the University of Illinois Health (UIH) IDOC telemedicine HIV clinic. Inclusion criteria were adult PWH under multidisciplinary care of UIH’s HIV telemedicine during custody from Jan 2021 - Oct 2024. If an individual was reincarcerated during the study period, each unique encounter was included. The primary objective was the incidence of PWH screened for TB in IDOC. Secondary objectives included incidence of LTBI / active TB, appropriateness of tx, incidence + mitigation of drug+drug interactions (DDI), & viral + immunologic function at intake vs release or completion of TB tx (whichever came first). Data collected included demographics, TB screening (IGRA), screening results, HIV RNA, CD4 count, HIV + TB tx regimens, & PharmD DDI detection + interventions.

**Figure 1:**
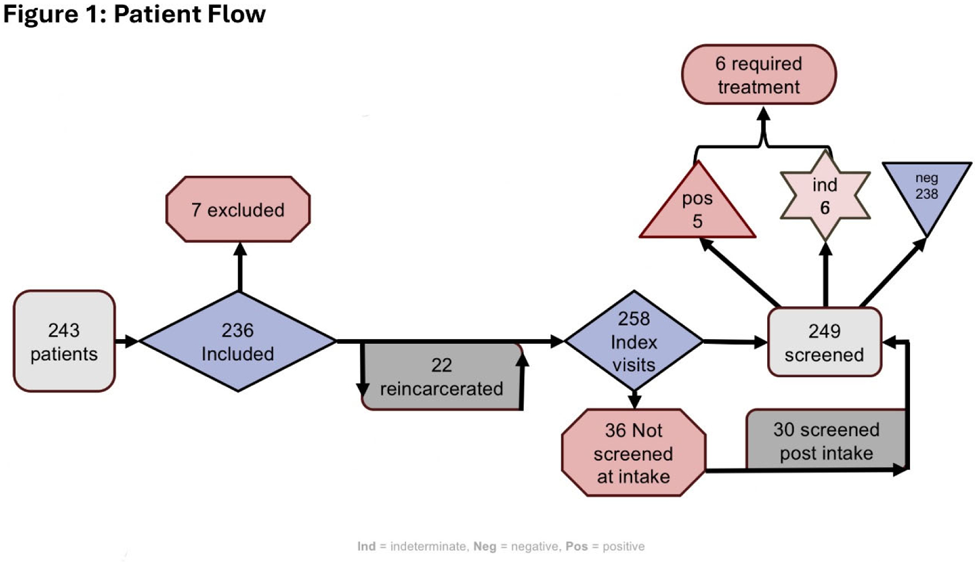
Patient Flow Figure 1 showcases the flow of subjects in the study. 243 were screened, 7 were excluded, 236 were included (22 of which were reincarcerated, leading to 258 intake visits where TB screening could again occur). TB screening did not occur at 36 intake visits, 30 of those subjects were screened at a later date by the request of the University of Illinois Hosptial’s (UIH) multidisciplinary telemedicine team, leading to a total of 249 tuberculosis screenings. 238 screens resulted negative, 6 indeterminate, and 5 positive. After medical history review & retesting by UIH’s multidisciplinary telemedicine team, only 6 of the 11 positive/indeterminate subjects required treatment for latent tuberculosis.

**Figure 2:**
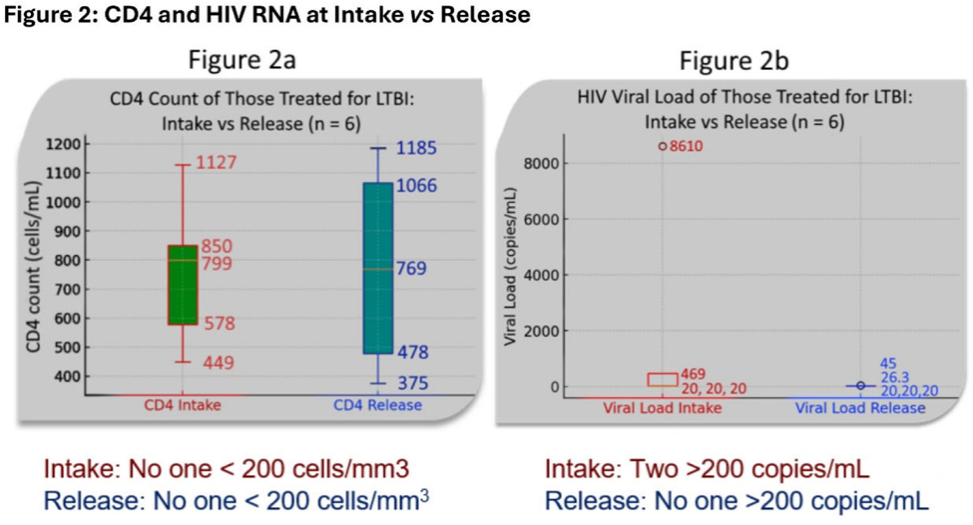
CD4 and HIV RNA at Intake vs Release Figure 2 showcases HIV outcomes (CD4 count, and HIV RNA viral load) of those treated for latent tuberculosis. The figure compares those subject’s labs upon intake to the Illinois Department of Corrections, versus their labs upon release. Figure 2a showcases CD4 count, and reveals that no patients had CD4 counts below 200 cells/mm^2 at intake or release. Figure 2b showcases HIV RNA viral load, and reveals that 2 subjects had uncontrolled viral loads at intake, and all patients were controlled upon release.

**Results:**

Of 258 encounters 91.5% were male, 76.4% were Black, with an average age of 37.5 years (Table1). TB screening occurred in 96.5% (249) of PWH in custody (84.9% occurring at intake). Of those screened, 95.6% (238/249) screened negative, 2.4% (6/249) indeterminate, & 2% (5/249) positive (Table2). No active TB cases were detected. Of the 11 positive/indeterminate tests, 6 required treatment for LTBI. Only 2 of which were guideline-appropriate; 83.3% (5/6) possessed DDIs, 100% of which were successfully mitigated (Table 2). At release all PWH treated for LTBI achieved CD4 counts >200 cells/mm³ (Figure2a) & viral suppression (< 200 copies/mL)(Figure2b).

**Conclusion:**

The study found high TB screening incidence & low LTBI rates with no active TB in PWH in custody in IDOC. The UIH Telehealth team played a major role in recommending TB screening post-intake, which may have otherwise been missed, & mitigating LTBI DDI; Favorable HIV outcomes were maintained during custody while receiving LBTI tx. A need for LTBI+HIV tx education was identified for IDOC intake team.

**Disclosures:**

Scott Borgetti, MD, GlaxoSmithKline: Grant/Research Support

